# Technological Barriers in the Use of Electrochemical Microsensors and Microbiosensors for *in vivo* Analysis of Neurological Relevant Substances

**DOI:** 10.2174/157015912803217350

**Published:** 2012-09

**Authors:** Bogdan Bucur

**Affiliations:** National Institute of Research and Development for Biological Sciences, Bioanalysis Center, 296 Splaiul Independentei, 060031, Bucharest, Romania

**Keywords:** Brain *in vivo* analysis, microelectrodes, microbiosensors, neurotransmitters, neuromodulators.

## Abstract

In this paper is presented an overview of the technological barriers faced by the in vivo brain analysis with microelectrodes. Numerous microsensors and enzymatic microbiosensors have been developed for the real time monitoring of neurotransmitters, neuromodulators, drugs and diverse other biological relevant substances. A clear understanding of the working principle, advantages and limitations is essential for the acquisition of valid data in neurological investigations. Some of the aspects presented here refer to: microelectrode insertion and positioning related to possibilities to minimize tissue damage, spatial and temporal resolution of the measurements, actual controversies in data interpretation and sensor calibration, simultaneous detection of multiple analytes, interferences and state of the art in the development of wireless devices.

## INTRODUCTION

Elucidation of biochemical processes is essential for the correct explanation of various unsolved questions in neuroscience, the ultimate goal being the correlation between changes in neurotransmitters and specific aspects of human behavior or medically relevant conditions. The *in vivo *analysis of neurological relevant substances, with spatial and temporal resolution relevant for single-cell monitoring, allows the investigation and eventual elucidation of different neurological processes (like learning, rewarding, synaptic transmission, etc.), pathological conditions and mental disorders (epileptic seizures, Parkinson, Alzheimer's disease, etc.), physiological reactions to trauma and effects of pharmacological treatments or illegal drug abuse. Chemical monitoring in the brain of freely behaving animals is important for assessing the validity of measurements collected under anesthesia [1] or in brain slices that can be grown for several weeks [2]. A large variety of techniques and equipment for brain investigation are described in literature: multielectrode arrays for deep-brain stimulation [3] or electrophysiological recording [4], functional magnetic resonance imaging-fMRI (more suitable for study of the effects than the biochemical causes), microdialysis (used for sample collection for *ex vivo *analysis) [5], various optical techniques ranging from fiber optics sensors [6] and NIR spectroscopy [7] to fluorescence microscopy [8] and microelectrodes (detailed in this review). The development of so many and various analytical techniques is explained by the interest of neuroscientists in more than 200 chemicals that act as neurotransmitters or neuromodulators (either physiologic or exogenous substances like drugs and psychotropic agents). Each one of the analytical techniques has specific advantages and shortcomings and the investigator must be familiar with the working principle of the equipment/technique in order to obtain maximum information and more important to avoid compromised results (interferences from the complex biological samples, spurious signals, analyzing outside the calibration range, sensibility drift during measurements, etc.). The more mature techniques are available and already used for *in vivo* investigations, but numerous alternatives currently exist only as proof-of concept demonstrated *in vitro* on biologically relevant samples (serum, blood, brain slices, neuronal cell cultures, etc.). The emerging analytical technologies are highly interesting because they represent the techniques that might be used in the future and the development of new equipment allows to obtain new information leading to scientific breakthroughs. 

This review is describing the recent advances and technical barriers still to be overcome in the development of electrochemical micro(bio)sensors for the analysis of neurologically relevant substances. The electrochemical analytical techniques have numerous applications in diverse areas including environmental, food, health, quality product and (bio)reactors monitoring and have been adapted and miniaturized for the analysis of different relevant substances in the brain. The development of chemical and biological microsensors for neurological analysis is based on development, adaptation and integration of electrochemistry, surface chemistry and enzyme immobilization techniques onto suitable microelectrodes. Electrode miniaturization has been possible based on advances in different fields ranging from electronics (for quantifying minute analytical signals), material science (for constructing miniaturized electrodes) and electrochemistry (for developing suitable analytical techniques) up to medical implants (for mitigating the fouling/degradation of the electrode surface due to antibodies, proteases, development of inflammation or scar tissues, etc). The development, optimization and characterization of microsensors must be done by analytical chemists in close collaboration with neurophysiologists to ensure a successful transfer of the technology from the *in vitro *proof-of concept to cell cultures and ultimately to *in vivo* analysis on animals and –eventually- on humans. Also, although the multielectrode arrays currently used in hospitals for brain stimulation or for electrophysiological recording for medical applications/physiological investigations are not bioanalytical techniques, they have allowed the improvement of microelectrode biocompatibility, study of the impedance [9], development of precise positioning techniques, study of their implant effect in anesthetized or freely moving animals etc; all this knowledge being transferable for microsensors development. 

Two critical disadvantages of the microelectrodes for *in vivo* brain analysis are: (1) the invasive effects of sensor insertion coupled with the constraints produced by the wires connecting to the signal recording equipment inducing stress and behavior modification and (2) the adverse reactions leading to inflammation of brain tissue that produces modification of the biochemistry in the sampled area and ultimately the development of a scar tissue that encapsulate the probe and prevent any useful data acquisition [10]. These two issues are aggravated by the fact that the total size of inserted electrochemical microsensor is substantially bigger than the micrometric size of the active surface of the indicator electrode that is usually underlined in the published papers. The microelectrode is inserted in a much larger capillary shaft and the overall sensor size is given by the three electrode system (indicator, reference and auxiliary). Like any measurement technique, microelectrodes must have sufficient selectivity and sensitivity for the desired analysis, qualities reached due to recent advances in electrochemistry (new electrode coating procedures, use of signal detection techniques like fast-scan cyclic voltammetry-FSCV, etc.). The electrochemical micro(bio)sensors are usually designed for single analyte detection, but there are examples of multianalyte detection: e.g. the simultaneous measures of choline and acetylcholine using mono and bienzymatic biosensors [11] or the detection of catecholamine and serotonin using two electrodes poised at two different potentials [12]. Besides the electroactive substances, different other analytes can be detected electrochemically by using enzymes (e.g. the real time detection of adenosine using three enzymes: adenosine deaminase, nucleoside phosphorylase and xanthine oxidase [13]) or ion-selective electrodes (e.g. the K+ detection [14]). Microelectrodes allow investigations with micrometric spatial resolution at subsecond response time and are interesting in many fields of neurobiological research like neurotransmitters release, pharmacological studies or biochemical investigations. They represent an interesting alternative to the analysis based on microdialysis which is suitable for investigation of the basal concentrations or is limited to concentration gradients that change in time rather than neurotransmitter bursts [15]. 

## TISSUE DAMAGE 

The insertion of a microsensor (like any foreign object- e.g. a microdialysis probe) into the brain produces a penetrating trauma leading to short term acute effects (like edema) and long term chronic effects (like infections, inflammation or gliosis leading to the formation of a glial scar). Monitoring and mitigating tissue modifications around an implant is essential for both acquisition of relevant analytical signals and for maintaining sensor functionality. Despite the precautions (reduced size of the implanted sensor, optimization of the surface biocompatibility) that should minimize the traumatic effects, various negative effects on brain tissue were reported such as decrease in neuronal and synaptic density at the edge of a probe extending up to 1.4 mm from the dialysis probe [16]. Besides microdialysis, knowledge about the impact of microimplants in brain was acquired from the various uses of microelectrodes: deep brain stimulation, brain–machine interfaces, peripheral nerve stimulation and analytical chemistry applications. The intimate knowledge of both short and long term effects of microsensor insertion is critical for each particular experiment set-up: for example the blood flow and glucose metabolism decreased near the microdialysis probe for the first two hours after implant, but recovered to nearly normal levels within 24 h [17].

The acute trauma depends on sensor size and insertion technique. The goal is to penetrate the pia mater without significant disturbing the brain tissue. The insertion technique (see details bellow) is aimed to minimizing the acute trauma effects, however one cannot avoid to damage the neurons in the path of the implant and to puncture the brain’s microvasculature and the blood–brain barrier (“kill zone”). Some of the acute effects of the implant are: diffusion of plasma exudate into the neuropil, perturbation of the natural pH buffering mechanisms, increased concentrations of extracellular neurotransmitters, serum proteins, ions and other solutes, irregularities in neuronal spike activity [18]. The blood–brain barrier usually heals after a few hours, but this is not always the case [15].

Some of the long term effects of implating microelectrodes into the brain are: neuronal cell loss in the close vicinity of electrode surface, the encapsulation of the probe by glial cells and the formation of an enveloping scar tissue, host immune response to a foreign body, protein adsorption on electrode surface (fouling) and infections. Improvement in biocompatibility of the electrodes and the support may mitigate some of these issues, but valid measurements over extended periods of time are still problematic. Most of the studies regarding the chronic effects of the implanted microelectrodes were performed with microelectrode arrays used for medical applications (neuronal stimulation and signal recording). However, some of the conclusions may be extrapolated for the microelectrodes used for biochemical analytical purposes with the mention that the micro(bio)sensors have a more complex surface than the bare metals and all the supplementary components (mediators, enzymes, selective membranes, etc.) can trigger immunological adverse reactions or might interact with different components of the extracellular fluids. Not only the chemical composition, but also the structure of the components used for microelectrode construction and/or surface modification may be important from the toxicological/inflammatory point of view, e.g. the carbon nanotubes have a certain toxicity while graphene does not interrupt the integrity of the blood brain barrier [19]. Different methods like histology, inflammatory response, immediate-early gene (IEG) expression, cytoskeletal integrity and apoptotic profiles were used to investigate the implant effects of multielectrode arrays that remain stable for more than 6 months [20], but micro(bio)sensors are not usually required to remain operational for such extended periods of time. In fact the operational life *in vivo *of the microsensors is substantially shorter, e.g. despite the fact that a glucose sensor maintains its response during 5 days of continuous use in buffered standard solution, if it is implanted for 8 hours (unused to allow the blood barrier restoration) the subsequent *in vivo *measurements are interpolated using a calibration graph made after extraction to obtain the remaining response [21]. Nevertheless there are remarkable microelectrodes capable to measure *in vivo *the concentration of dopamine for up to 4 months by using a conventional carbon-fiber microelectrode, but inserted in a polyimide-coated fused-silica capillary that is both highly biocompatibile and less rigid [22]. Besides tissue reaction to the implanted device, the operational stability of the inserted microbiosensors might be affected also by issues related to sensor fabrication like enzyme instability or mediator loss. In consequence, it is essential to properly test the microbiosensors not only in buffer solutions, but also in conditions as close as possible to the biological medium (37°C, continuous operation, presence of proteases, inhibitors, free radicals, etc.).

Fixing in place the microelectrodes has an important long-term influence on brain tissue. In comparison with freely moving microelectrodes (wireless, unconnected), the tethered microelectrodes produced significantly greater reactivity of the antibodies that is not due only to the presence of bone screws and fixing resins. The persistence of macrophages surrounding the fixed implanted devices has deleterious effects on adjacent nerve cell. Thus, after four weeks, the decrease of the neurofilaments around tethered implants was important, while only little loss was observed for the untethered electrodes leading to decrease of electrode performances [23]. One way to mitigate this issue is the use of flexible polymers for the construction of a microelectrode array that allowed the neural recording during 60 days in awake, behaving rats. The signals had undiminished amplitude and signal-to-noise ratios for 8 weeks after chronic implantation due to the minimal tissue [24]. A mechanically adaptive polymer was used as support for microelectrodes. The material was initially stiff to minimize the trauma produced by the penetration through the pia mater and to facilitate insertion into the cortex. Subsequently the material had the ability to morph while in the cortex to match more closely the mechanical properties of the cortical tissue and thus it was possible to minimize the forces exerted on the tissue and to attenuate the inflammation. After eight weeks it was observed a lack of tissue necrosis or excessive gliosis at the microelectrode-tissue interface [25]. 

## MICROELECTRODE INSERTION AND POSITIONING 

The insertion of microelectrodes aims to the precise positioning of the sensors in specific places of the brain with high spatial resolution and minimal tissue damage. The same analyte might come from multiple sources such as the bloodstream, non-neuronal cells or cells sectioned during implantation and in consequence the exact location of the active surface of the microelectrode is very important for the correct data interpretation. The presence of a neurotransmitter in the synaptic gap between two neurons is correlated with direct transmission (neuron to neuron). If the neurotransmitter reaches the extrasynaptic space (e.g. by direct release or escaping the synapse) then the signal may be transmitted to other distant neurons. The required spatial resolution of the analysis is given by each particular investigated phenomenon. Some of the smallest desired sizes are given by the synaptic gap that is under 50 nm or the neurotransmitters releases from vesicles, but the working electrodes usually have a length between 50 and 200 μm extended beyond the glass insulation and a diameter in 1-50 μm range. One of the smallest reported electrode/biosensor was obtained by the attachment of a multiwall carbon nanotube to the end of an etched tungsten tip. The reported sizes are 30 nm for diameter and 2-3 μm for length [26]. Monitoring of different biological phenomena like vesicular exocytosis or oxidative stress was successfully achieved using microelectrodes for single cell cultured *ex vivo* [27], but these performances are not easily extended to *in vivo* measurements in brain [28]. Nevertheless, a small size is not always an advantage, e.g. the glucose analysis in extracelular space is made with microelectrodes substantially larger than capillary and thus the concentration detected is relevant for local average extracellular glucose level and not falsified by a heterogeneous diffusion from the capillaries [21]. On the other hand, if the microelectrode is too large then it might detect anomalously high concentrations of some analytes. For example, the detected concentration of uric acid in the extracellular fluid was reported to be ~50 times greater when detected with 320 µm diameter carbon paste electrode in comparison with the concentration detected using a 160 µm diameter electrodes, but the concentration of 5-hydro-xyindoleacetic acid was similar for 320, 260, and 160 µm diameter electrodes. The authors speculate that large electrodes perturb the surrounding tissue and induce the production of uric acid [29].

The exact location of the microelectrodes may be established by post-mortem histology. Besides the brain tissue staining, it is necessary to produce lesions at the end of the experiment either using a high current (that destroys the microelectrode and prevents post-extraction recalibration of the sensor) or with a tungsten microelectrode (a non-destructive approach) [30]. This technique has obvious shortcomings and real time non-invasive alternatives are highly useful both for guiding and confirming micro-electrode’s placement. Among such alternatives one should mention X-ray (with or without contrast substances), computer tomography (also X-ray detection, but with 3D spatial resolution), ultrasonic techniques or MRI. The most important problems that need to be solved with any imaging techniques are: the establishment of the optimum and precise location in which the microelectrodes should be inserted and verifying that the microelectrodes were correctly positioned (e.g. deep enough to be in the desired area). Imaging techniques can confirm if electrode insertion “hit” or “missed” the desired target brain region based on physiological and/or anatomical criteria and in consequence can provide invaluable information about the validity of the measurement [31]. Color Doppler imaging allows the monitoring of the precise position of the microelectrode during the insertion in the brain of alert monkeys. The accidental injury of the blood vessels by the microelectrode can be avoided using the detailed images of the cerebral cortex provided by this technique [32].

**Fig. (2):**
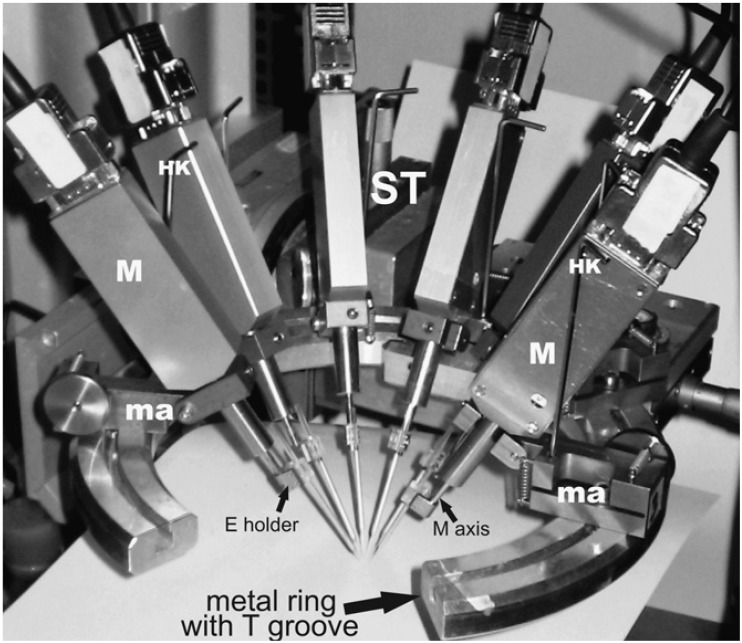
Independent positioning of six microelectrodes for multisite recordings *in vitro*. The manipulators (ma) holding the micrometers are mounted on a metal ring by a screw and nut fixed in a T groove. M, micrometer; HK, hex-key; M axis, axis of micrometer; E holder, microelectrode holder mounted on M axis; ST, solid xyz-stage for coarse positioning of position system. Reprinted from Journal of Neuroscience Methods 176, 182–185 (reference 33), Copyright (2009), with permission from Elsevier.

The implantation equipment range from computer-controlled micromanipulators to manual insertion, the mechanic micropositioner being based on hydraulic, pneumatic, spring-loaded or other motorized microdrives. One example of a micromanipulator used for the insertion of an array with 16 microelectrodes with electrochemically grown hydrous iridium oxide for the potentiometric detection of pH is presented in Fig. (**1**). A plastic cortical cup containing the reference electrode and a buffer solution used for microelectrode pre and post measurement calibration was fixed to the skull of anesthetized rats around the craniotomy with a bone screw and dental acrylic [18]. There was also described a possibility to position and insert independently several microelectrodes to investigate different areas of the brain (Fig. **2**) [33]. The magnitude of the insertion speed used by different research teams decreases from fast movement of 1-8 m/s [34] to moderate speed ~2 mm/s [35] and to slow motion 5 µm/s [36]. The movement may also have variable speed, pulsations or push-partial withdraw cycles. Using a pH sensitive microelectrode made from iridium oxide deposited on a silicon-substrate it was demonstrated that insertions at 1.0 mm/s were less traumatic than 50 µm/s (shorter acidosis) [18]. The probe insertion with a vibrating impact frequency close to the resonant frequency of the material is effective in reducing deformation [37]. Another possibility to reduce by up to 40% the peak insertion force is the pre-treatment of the pia mater with collagenase [38].

## FABRICATION ISSUES

There are several fabrication constrains that the microelectrodes must fulfill. It is obvious that any fracture of the material poses a great risk for the patient’s life. The reliability and safety of the implanted devices in highly sensitive organs must be sufficient to prevent any catastrophic fracture to be applicable on human subjects. Usually the microelectrodes were deposited on silicon support because this technology was borrowed from microelectronics devices, but silicon is a brittle material. Titanium provides a better alternative to silicon for the development of robust penetrating microelectrodes [39], but it must be remembered the flexibility requirement for minimum tissue damage (see above). 

A classical electrochemical cell consists of three electrodes: working electrode (WE, indicator, the one that provides the analytical signal), the reference electrode (RE, it has the same potential in different working environments) and auxiliary electrode (AE, counter electrode, made from inert material with larger area than working electrode used to pass the current from WE without disturbing the RE). A two electrode cell (WE+RE) with smaller dimensions is often used for *in vivo* measurements. The two electrode configuration is considered by electrochemical analysts to be less desirable due to the fact that the current passage through the RE usually leads to variation in time of the reference potential. The currents recorded using microelectrodes are very low (pA to nA) and should not negatively affect a normal size RE, but this assumption may not be valid for micrometric sized RE. The multielectrode arrays made by vapor deposition should favor the use of three electrode configuration by simply using one of the electrodes as AE instead of WE. The geometry of the electrochemical cell is also important, e.g. a long distance between the WE and AE leads to an ohmic drop (or IR drop) due to electrical resistance of the measurement sample. Multiple WE may be used simultaneously or sequentially with the same RE and AE each one with the same potential or with different potentials (e.g. one WE with the immobilized enzyme to detect the analyte together with the interferents and the second WE without the enzyme to evaluate only the interferents [40]). 

Usually the papers give details only concerning the development of the proposed/employed WE, but the analytical performances are given by the entire electrochemical cell composition and configuration. The most used RE is Ag/AgCl electrode that can be a classical RE with internal solution in a glass shaft used for *in vitro* experiments or reduced to only a silver wire covered by AgCl fixed on the capillary that holds the WE [41]. An unprotected Ag/AgCl wire inserted *in vivo* suffers rapidly an important shift in potential leading to sensor failure due to two phenomena: the AgCl dissolution due to the complexation of Ag^+^ and the formation of an inflammation tissue around the electrode due to the poor biocompatibility of AgCl. The extension of the operational live of the RE inserted subcutaneously in rats can be extended up to two week by coating the electrode with a membrane of thermally cured Nafion or polyurethane that will both reduce the dissolution of the AgCl layer and substantially improve the biocompatibility. Both polymer coatings do not prevent transfer of ions to the Ag/AgCl electrode and thus good current/voltage characteristics are obtained for the coated RE, but this implies that Ag^+^ and C1^-^ are transported across the coatings and does not explain the mechanism of stabilization [42]. Another study demonstrated that, while uncoated Ag/AgCl RE lead to the shift of a dopamine peak potential of 200 mV, Nafion coated wire RE Ag/AgCl may be used for up to 28 days. The authors explain the better performances of the coated RE by small differences of the scar tissue: the lesion around bare electrodes have a rough structure that implies that the cells are strongly adsorbed on the bare surface leading to the formation of a heavy buildup of organic plaques. By impedance spectroscopy it was demonstrated that there are no differences between coated and uncoated RE and in consequence the potential shift was attributed by authors to a microenvironment around the bare RE that alters the chloride equilibrium that is avoided by Nafion [43]. In order to minimize the local tissue damage in the analyzed area, the RE may be implanted at a remote site of the brain, e.g. in the opposite hemisphere from the recording areas [44]. Different pseudo RE (e.g. the screw that fix the skull bones [45]) may be used but with important precautions: they do not have a “known accepted” value of their potential, their reference potential may vary from one sample to another or drift during the experiment, the reported potentials are not easily comparable with other researchers, etc. In fact, from an electrochemical point of view, even a silver wire coated with AgCl is considered to be a pseudoelectrode because it does not have an internal standard solution and its potential is dependent on the Cl^-^ concentration, however it is used *in vivo* on the assumption that the local Cl^-^ concentration is constant during the experiments and the physiological Cl^-^ concentration is similar in different subjects. The dependence of potential of RE without internal solution on the Cl^-^ concentration was address for screen printed electrodes by using membranes that contain a known concentration of KCl [46]. A liquid-junction Ag/AgCl reference electrode was microfabricated by covering the electrode with a polyimide protecting layer that has a 50-μm-wide slit. The RE maintains its potential for more than 100 h with insignificant dependency on external KCl concentration and pH [47].

The microelectrodes used for chronic measurements may be affected in time by numerous processes like fouling, corrosion, mediator leakage, enzyme denaturation. These negative phenomena must be investigated and mitigated for each particular microelectrode developed. *In vitro* optimizations and measurements may not be easily extrapolated to i*n vivo *conditions where synergistic and antagonistic effects are observed. Thus, the corrosion rate of tungsten microelectrodes measured *in vitro* in phosphate buffered saline solutions saturated with oxygen or containing 30 mM of hydrogen peroxide was higher than *in vivo*. The authors explained this unexpected improvement of microelectrode characteristics based on correlation of the corrosion with the concentration of the reacting species in the cathodic reaction (oxygen and hydrogen peroxide) that are prevented to reach the microelectrode surface by the formation of a biofilm [48].

## SPATIAL RESOLUTION 

One advantage of the microelectrodes is the small area investigated. The spatial resolution of the analysis depends on the size of the microelectrode and the diffusion of the analytes from bulk solution/tissue to the electrode surface. Multiple electrode arrays may be used in order to simultaneously investigate the same or different analytes in different brain regions situated in close proximity (instead of a single electrode implanted in different animals). Two aspects must be taken into consideration for a successful application of the electrode arrays: the prevention of electrical crosstalk between the electrodes and the use of a spatial separation between the electrodes, large enough so that each electrode measures independently. It is important to make the difference between the electrical crosstalk (current leaking between microelectrodes) and chemical crosstalk (analytes diffusion from one electrode to another leading to an overlap of the diffusion layers). Both phenomena have a negative impact on the spatial resolution and depend on microelectrodes size and spacing and distance to investigated cells. The electrical crosstalk depends also on the electrode impedance and electrical signals characteristics. The investigation of chemical crosstalk is based on mathematical models that describe analyte transport by diffusion from neuron to the electrode surface, based on the diffusion coefficients and the geometry of the investigated tissue and electrode. Besides the ratio between the microelectrode size and the distance between different microelectrodes from the array, there are numerous other supplementary phenomena that must be taken into consideration like the analyte reaction with oxygen (that may be faster due to the electrode insertion), analyte consumption at the electrode (continuous in the case of amperometry and in short bursts for FSCV) [49].

Two and four independent carbon fiber working electrodes with a diameter ~ 1 µm were constructed in the same capillary and used for the dopamine analysis. Different spatial resolutions were investigated and, only for two electrode configuration it was observed that the responses were independent and provided information on different dopamine concentrations when the electrodes were 10-15 µm apart. However, when the electrodes were closer, they behaved as a single unit rather than individual electrodes in a bulk solution. This suggests that the two elements are able to monitor local dopamine concentrations at distinct, but closely spaced, recording sites. In contrast, the attempts to produce four-microelectrodes resulted in fused geometry that was not able to provide data with spatial resolution [50]. Subcellular heterogeneity in single-cell exocytosis was electrochemically detected with a multielectrode array (10−20 μm) composed of individually addressable 5 μm diameter microdisks with embedded in the same glass capillary [51]. Such a small distance between the electrodes raises questions if the electrodes really operate independently due to the requirement to insure that the chemical diffusion length in solution is much longer than the distance between electrodes. Dopamine was monitored *in vivo* at spatially different brain locations using a multielectrode array made with four working carbon fiber microelectrodes each one separated by 250 μm and at this distance the recorded responses were independent [30]. 

The requested spatial resolution depends on each particular investigated phenomenon, but a micrometric specificity is not always necessary. There are cases when a spatial resolution between electrodes is not even wanted, e.g. differential measurements one with the enzyme immobilized on the surface to measure the analyte and the interferents and the second sensor lacking the enzyme to obtain only the background signal [52]. 

## CONTROVERSIES IN DATA INTERPRETATION AND SENSOR CALIBRATION

Interpretation of the analytical signals obtained from measurements made *in vivo* with microelectrodes is not always easy and straight forward. The direct correlation of the results obtained with the same microelectrode in standard solutions with the measurements *in vivo* is prevented by several aspects. Firstly, the mass transport of the analytes to the microelectrode surface is different (in standard solutions transport occurs by diffusion and convection while *in vivo *there are membranes and biofilms that drastically reduce the analyte flow) [49]. Secondly, there are numerous and sometimes unexpected interferences from the complex sample matrix that can be only partially investigated *in vitro* [11]. One should mention also the limited operational stability of the sensors in biological media [21]. Also, attributing the signal to the analyte (and not to an interfering parasite signal) is difficult because the total quantity of the measured substance in such tiny volumes is very small, the recorded currents are extremely low (proportional with the electrode surface) and any noise/spike will be superposed over the recorded data. Besides the technical limitation of the microelectrodes, the results may be compromised due to experimental factors like: tissue damage&modification due to sensor insertion, behavior modification and stress induced by surgery and measurement constrains, effects of anesthesia on brain chemistry, some analytes might come from multiple sources (e.g. bloodstream or non-neuronal cells) [10]. All these factors prevent an easy interpretation of the analysis results and in consequence, special care is given to the correct elucidation of the biochemical phenomena. For a greater confidence in the analysis results, different analytical techniques may be used for the detection of the same compound or external stimulus with known consequences may be applied. Interpretation of the analytical signal is facilitated by the external induction of analyte variation and monitoring of the subsequent expected evolution (e.g. glucose monitoring after glucose or glucose & insulin injection [21], dopamine concentration increase by unexpected food delivery [22] or locally infusion of ascorbate with a microdialysis probe [53]). In fact is easier to monitor the variation of analyte concentration in time than the exact analyte concentration. 

There are different strategies for optimum sensor calibration: pre and postcalibration *in vitro*, calibration in solutions made with a composition comparable with the measuring media and comparison of the results with another analytical technique. The microelectrodes results are compared with a microdialysis probe, but differences between the two alternatives might be produced by the larger microdialisys probe (with different spatial resolution and more important tissue damage) or by difficulties in microdialysis calibration [15]. The detection with a carbon fiber disk microelectrodes by fast-scan cyclic voltammetry (FSCV) of N-acetyl-p-aminophenol injected intraperitoneal was compared with microdialysis in order to determine which *in vitro* calibration procedure (precalibration or postcalibration) provides the most accurate results. It was observed that the electrochemical results were approximately two times greater for postcalibration and smaller for precalibration than the microdialysis result [54]. It is important to make the *in vitro* calibration in solutions that contain the same interferents with the sample matrix. Dopamine analysis with carbon-fiber microelectrodes provides double responses in buffer solution in comparison with artificial cerebrospinal fluid that contains calcium and magnesium ions. This phenomenon is explained by the fact that the negatively charged carbon-fiber electrode surface attracts both the positively charged analyte and other amine metabolites and in consequence the presence of cations modifies the selectivity ratio [55].

All the factors listed above explain the difficulties of all analytical techniques to determine the basal analyte concentration* in vivo*. Besides the difficulties of the detection *in *vivo, each analytical technique has specific constrains, e.g. the FSCV is a differential technique that subtract background charging current from the recorded signal and can only provide information about analyte concentration fluctuation and not estimation of the concentration. The analytical signal produced by variation of analyte concentration is relatively small in comparison with the background current. In consequence, it is necessary to know the exact value of the background (determined at the basal analyte concentration). In these conditions any background drift/modification is preventing a meaningful data inter-pretation due to signal distortion. In practice, the background drift is so important that FSCV measurements are possible only during short intervals (less than two minutes). The experimental working parameters (waveform, cycling, data collection, potential range, etc.) must be optimized for each particular case for a reduction of the rate of background drift without sacrificing analysis sensitivity. A special measurement procedure termed “analog background subtraction” was developed in order to minimize negative impact of the background. This procedure consists in playing back of the inverse background during data acquisition in order to cancel the background in measured scan. The authors reported a reduction of the background drift that allowed the monitoring of evoked release of dopamine during 15 min in the brain of an anesthetized rat and even dopamine monitoring in the brain of freely moving rats for up to 30 min [56]. 

## MULTIPLE ANALYTE DETECTION 

Electrochemical detection methods have relatively limited ability to detect multiple analytes, especially in comparison with the other alternative developed for brain biochemistry analysis such as sample collection with microdialysis probes and analysis based on chromatographic or electrophoretic separations. The main interest of microelectrodes consists in their ability to achieve high spatial and temporal resolution, but some electrochemical analytical methods that have been developed are able to simultaneously detect several analytes. Sweeping techniques like FSCV make the measurements in a potential domain and in consequence they might detect several electrochemically active substances if their redox potential is sufficiently separated. Another option is the use of different multi-electrodes, each one optimized for the detection of a single analyte, but this strategy requires multichannel detection using with more expensive potentiostats. 

**Fig. (3):**
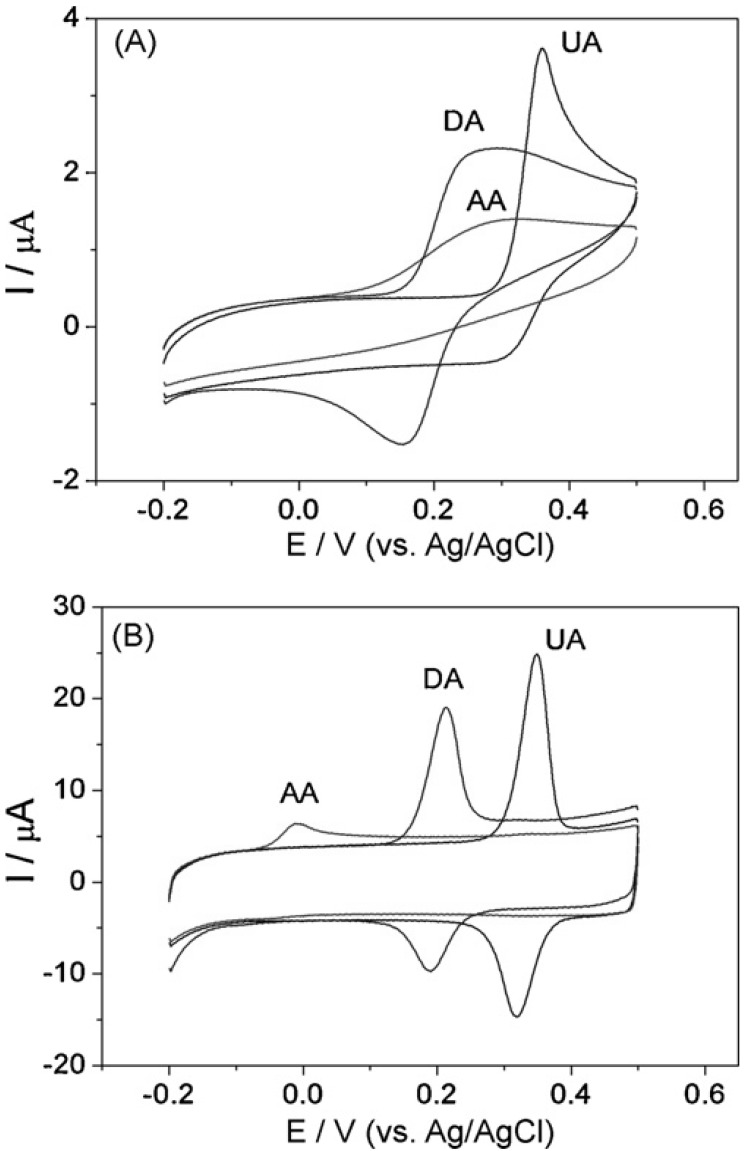
Cyclic voltamograms of uric acid (UA), dopamine (DA) and ascorbic acid (AA) at (A) bare glassy carbon electrode and (B) carbon nanohorns modified electrode. Reprinted from Biosensors and Bioelectronics 25(4), 940-943 (reference 57), Copyright (2009), with permission from Elsevier.

The development of potential sweeping analytical techniques able to simultaneously detect different electro-chemically active neurotransmitters is based on electrode surface modification that assures both high electrocatalytic activity (high current) and peak separation (different redox potential). For example, the voltammograms obtained using bare glassy carbon electrode for dopamine and ascorbic acid are superposed and close to uric acid, but electrode modification with single-walled carbon nanohorns allowed the separation of these analytes (see Fig. **3**) and sensitive multianalyte detection by linear sweep voltammetry (LSV) [57]. The use of unmodified edge plane pyrolytic graphite electrode (a material that contains both basal plane and edge plane surfaces with different electrochemical properties) allowed the simultaneous determination of dopamine, serotonin and ascorbic acid by differential pulse voltammetry [58]. Among other electrode modification methods are the deposition of Nafion, poly-phenylenediamine, phthalocyanine, DNA, nanoparticles, organic redox mediators, various carbon nanotubes, etc. It is not always necessary to obtain a separation of the peaks potential. Thus, epinephrine and dopamine were simultaneously determined using gold electrodes modified with 2,3-dimercaptosuccinic acid based on the differences between the anodic and cathodic behavior: the cathodic peak currents depended linearly only on the concentration of dopamine, while the anodic peak currents were equal to the sum of individual anodic peak currents of epinephrine and dopamine [59]. 

Unlike FSCV, the linear sweep voltammetry and differential pulse voltammetry are slower analytical techniques. In FCSV, the voltage is cycled at high rates (typically >100 V/s), much faster than classical sweep techniques (usually < 100 mV/s). FSCV usually employs bare electrodes because they have faster electron transfer and less signal dampening than modified electrodes [60]. Nevertheless, the minimum analysis time depends on both the sweep time and the time taken for the concentration of analyte near the electrode to return to its unperturbed value by diffusion through the tissue matrix [61]. Although possible, the simultaneously detection of more than one neurotransmitter by FSCV is challenging due to signal shape alteration produced by background subtraction. The discrimination between dopamine and serotonin (two neurotransmitters with similar electrochemical properties) in the presence of high concentrations of ascorbate (a common interferent) was possible by combination of appropriate large-amplitude/high-frequency voltage excitation in specific regions in the voltage space (voltage windows) with signal processing techniques valid for the analysis of nonstationary and nonlinear phenomena [62]. Another study reports the simultaneous detection of serotonin and histamine without interference from dopamine based on the difference in the peak position (potential) of the two analytes [63]. The simultaneous analysis of several substances that give overlapping cyclic voltammograms may be achieved by chemometry using statistical data interpretation like principal component analysis (PCA). The induced changes in dopamine and pH in a region of a brain that contains dopaminergic terminals were successfully resolved using a set of 30 cyclic voltammograms recorded for 9 substances at different concentrations [64]. Nevertheless, PCA can be used only when known species contribute to the signal and any substance that was not used for calibration may cause distortion of the voltammograms. Another proposed strategy is the use of “paired pulses” (the fast CV scans are separated by different time gaps) leading to the recording of two different voltammograms. The peak oxidation/reduction currents are correlated with two time dependent phenomena: analyte transport from solution to the electrode surface and subsequent adsorption. The monitoring of three different analytes (dopamine, adenosine and pH) was possible based on the specific kinetics of these phenomena corresponding to each analyte and the variable time periods between FSCV [65]. 

Combining multiple electrodes allows the measurement of different analytes. The main advantages of multielectrode arrays are the possibility to reduce the number of testing animals (more analytes detected in the same experiment), the simultaneous monitoring of analytes variation produced by the same external stimulus (e.g. glutamate and acetylcholine peaks induced by KCl injection with a micropipette attached to the microelectrode array [66]) and the possibility to improve the measurements reliability by using a biosensor coupled with an electrode without enzyme to measure the interferences or multiple similar biosensors to test the reproducibility [67]. Another interesting application of the multielectrode approach is the possibility to simultaneously detect the same analyte in different location of the brain (multisite monitoring) [30]. The multielectrode approach may seem a simple combination of different working electrodes each one selective for a single analyte, but the reality is more complex and each particular *in vivo* experiment demands a thorough understanding of both the analytical device and investigated neurological phenomenon. For example two identical carbon-fiber microelectrodes may be used for the detection of dopamine and norepinephrine by FSCV. Despite the fact that these two analytes have similar electrochemical properties and cannot be distinguished based on their voltammograms, the electrodes were inserted in different brain regions where only one compound is present: dopamine in anterior nucleus accumbens (NAc) and norepinephrine in the ventral bed nucleus of the stria terminalis (vBNST). The authors confirm this specificity with the aid of pharmacological manipulations that alter the level of only one catecholamine while the electric stimulation induces simultaneous release of both dopamine and norepinephrine [68]. Another interesting application is the simultaneous use of biosensors one based on cholinoxidase (for the detection of choline) and the second based on cholinoxidase and acetylcholinesterase (for the detection of acetylcholine together with choline). The system was used to test if the released acetylcholine is completely hydrolyzed by endogenous acetylcholinesterase before reaching in the extracellular space. The authors found that both biosensors gave the same signals when acetylcholine release was induced by a robust evoked stimulus (KCl) or a less potent and more physiological stimulus (nicotine). This is a clear indication that acetylcholine does not spill over into the intracellular space and biosensors based only on choline-oxidase should be enough for the correct measurement of synaptic acetylcholine release. The authors explain the published contradictory reports by variation across different microelectrodes placements, irreproducibility between microelectrodes or measurements artifacts caused by physical manipulations and recommend bielectrode system one coated with cholinoxidase and the second with an inert protein to asses these spurious signals [69]. The bienzymatic detection of acetylcholine in presence of choline is questionable because the choline concentration *in vivo* is substantially higher than acetylcholine and even a small error in choline measuring will induce unacceptable high errors in acetylcholine detection.

## INTERFERENCES

The numerous potential interferences found in the highly complex biological media sampled during *in vivo* investigation pose obvious problems for any analytical method. While the microdialysis sample extraction coupled with analysis after separation by electrophoresis or chromatography tends to be less affected by interferences, the electrochemical detection is made without any pre-separation and in consequence the interference issue has a crucial influence on the validity of the results. There are several alternatives for interference mitigation applicable for electrochemical detection: electrode modification with electrochemical mediators to reduce the applied over-potential, the use of selective membranes to cover the electrode surface, the use of blank electrodes made without enzymes to record the background current, etc.

Electrode modification with electrochemical mediators reduces the applied overpotential required for the analyte oxidation/reduction to the potential of the mediator, e.g. hydrogen peroxide detection with bare Pt electrodes is made at +700 mV while polyaniline (PANI) measures at -300 mV (in reduction) and Prussian blue mediator decreases the working potential to ~0 mV. Please note that it is important the absolute value of the applied overpotential (e.g. -300 mV is bigger than 0 mV) measured against the reference electrode and not the open circuit potential of the working electrode in the sampled media. The modified electrodes are less affected by interferences, but one has not to forget that a mediator might interact not only with the analyte but also with other electrochemically active substances from sample matrix, e.g. PANI mediated detection of hydrogen peroxide is perturbed by oxygen and the solutions applicable *in vitro* like purging with nitrogen or scavenging with sodium thiosulfate [70] are not useful for *in vivo *analysis. Prussian blue as a mediator has another disadvantage: it is not stable at neutral and basic pH and different membranes are used to improve the stability. Some of these membranes, like Nafion or poly-phenylenediamine (PoPD), have also the additional advantage that improve the electrode selectivity towards interfering substances found in brain (ascorbic acid, 3,4-dihydroxyphenylacetic acid, dopamine and uric acid) [71]. 

The use of membranes for the minimization of interferences is based on different interactions between membrane and interfering substances in comparison with membrane and analyte. Microporous membranes like cellulose acetate separate only the large interferents from small analytes [72]. One of the most common electrically charged membranes used for electrode modification is made from Nafion that is a polymer of sulfonated tetrafluoroethylene. Nafion is a cation exchange polymer with pKa =5.45 that can be modified by incorporating of different substances, e.g. acridine changes the apparent ground-state pKa to 9 [73]. At physiological pH, Nafion-coated surfaces are effective in reduction of the interferences produced by anionic compounds (e.g. ascorbic acid, uric acid, etc), but they are attracting the cationic species (e.g. dopamine, serotonin, epinephrine, norepinephrine, etc.) [74], while the neutral substances (e.g. hydrogen peroxide) are slowed only by the diffusion through the film. Besides only partial interferences segregation, Nafion has additional drawbacks (non-uniform thickness and poor reproducibility) and was replaced in the construction of modified electrodes selective to hydrogen peroxide by poly(o-phenylenediamine) or overoxidized polypyrrole films that have lower permeability to both cationic and anionic interference due to different exclusion mechanisms (size and/or charge exclusion) [74]. The immobilization of l-glutamate oxidase in a silica sol-gel on platinum microelectrode modified with a layer of poly(phenylene diamine) allowed the construction of biosensors for L-glutamate with excellent selectivity towards L-glutamine, L-aspartic acid, L-ascorbic acid, dopamine, uric acid, serotonin and catechol while using a high overpotential (600mV vs. Ag/AgCl) [75]. The perm-selectivity of poly(phenylene diamine) layers can be enhanced by inclusion of globular proteins or β-cyclodextrin [76]. In Fig. (**4**) is presented a combination of two membranes. On the surface of the platinum electrode is deposed first a Nafion membrane for interference removal followed by an enzymatic layer (L-lactate oxidase for L-lactate measurements in this particular case). In the end, a supplementary membrane of polyurethane is deposited to reduce the amount of analyte that diffuses towards the enzymatic layer in order to obtain a linearity range of the biosensor compatible with high lactate physiological levels in brain (the sensor response is controlled by the mass transport of the analyte) [67]. Microelectrodes are not always covered by membranes just to minimize the interferences, an improved biocompatibility may also be obtained. Thus, bare platinum microelectrodes used for oxygen detection are poisoned after insertion in the brain, but a microelectrode covered with poly(phenylene diamine) showed only a minimum change after 3 days exposure to brain tissue [77]. Some membranes may provide the required selectivity for measurement in biologically relevant samples, e.g. glucose is usually detected with enzymatic microbiosensors based on glucose oxidase to take advantage of the enzymatic selectivity towards substrate, but nickel microelectrodes covered with a poly (sulfosalicylic acid) film may be used for glucose analysis with minimum interferences from ascorbic acid and uric acid [78]. All permselective membranes increase the response time due to the introduction of a diffusion layer on the sensors surface and have only limited interferences removal capacity (if the concentration of interferent is much higher than the analyte then an interfering signal is recorded).

Differential measurements are made between two electrodes poised at the same potential: one enzymatic biosensor to record the analytical signal and the background signal produced by interferences and a second electrode produced using an inert protein instead of the enzyme to record only the interferences [52]. The use of membranes for partial removal of the interferences is still employed in differential measurements in order to decrease the background signal to the minimum. Even when the membrane of poly(phenylene diamine) allows a rejection of interferences produced by dopamine and ascorbic acid close to 100%, some authors used differential measurements made with two working electrodes (one biosensor modified with enzymes like choline oxidase or L-glutamate oxidase and the other one a blank with inert BSA) in order to completely eliminate interferences and to substantially reduce external influences such as electrical noise or artifacts [79]. The differential measurements are not always performed between a biosensor and an identical electrode without enzyme. The interferences produced by H^+^ and Ca^2+^ ions at the detection of catecholamine by FSCV with a carbon fiber electrode were estimated using two ion selective electrodes to monitor pH and Ca^2+^ [80].

It is important to study not only the interferences produced by the substances usually found in brain, but also by the drugs used in the experiments to induce analyte variation. Thus, it was observed that GBR 12909 and nomifensine (two commonly used dopamine reuptake inhibitors) drastically reduce the sensitivity in the analysis of dopamine made with carbon-fiber microelectrodes modified or not with Nafion [81]. A similar study was done for norepinephrine detection with carbon fiber and diamond microelectrodes and was observed that cocaine, idazoxan and PPADS (pyridoxal-phosphate-6-azophenyl-2',4'-disulfonate) are electrochemically inactive at both microelectrodes at the potentials used for norepinephrine analysis. On the other hand, the norepinephrine detection in the presence of prazosin or capsaicin is complicated by the fact that both drugs are oxidized at similar potentials like norepinephrine while yohimbine and UK 14,304 reduced the recorded currents [82]. These studies demonstrate that it is important to investigate the influence of the drugs on electrode response, sensitivity and/or stability.

## OTHER ISSUES 

One important factor is the origin of the detected analyte: neuronal, astroglial, blood vessel, synaptic or extrasynaptic. In contrast with the microdialysis probes that are usually too large and with a long response time to detect exocytotic release, the microelectrodes with improved spatial and temporal resolution seem more appropriate for this investigation. Thus, the glutamate detected using microdialysis in basal conditions is mainly resulting from non-synaptic sources, but after external stimulation a significant part of glutamate in the microdialysate solution might be derived from neurotransmission. On the other hand the microbiosensors are more likely to respond to glutamate corresponding to basal synaptic events [83]. 

The response time of the microbiosensors produced by enzyme entrapment in different microporous matrix may be relatively long (up to 10 sec) to observe the vesicle release of neurotransmitter. To avoid the need of the slow diffusion of analyte inside the coating matrix, one alternative is to immobilize the enzyme directly on the electrode surface. Usually, simple enzyme adsorption does not produce stable biosensors due to biocomponent leakage, therefore binding groups (e.g. amino, carboxyl, etc.) are required on the electrode surface. One general method to functionalize different electrode materials is based on aromatic diazonium chemistry, a relatively instable compound produced *in situ* that can be used for the electromodification of the electrode. One key aspect of the diazonium based electrodeposition of aryl groups is the tendency to produce multilayers that passivate the microelectrode and in consequence the biosensor fabrication must be thoroughly optimized using electrochemical techniques for surface characterization like electrochemical impedance spectroscopy or cyclic voltammetry [84].

The chemical properties of the analyte in standard solutions used for calibration and in sampled tissue are highly important, because some substances are unstable. For example, nitric oxide (•NO) is an unstable compound produced within the cell that acts as neurotransmitter or vasodilator and is involved in neurodegeneration. In presence of oxygen •NO is spontaneously transformed into nitrite (NO_2_^−^) and both compounds can be oxidized at high potentials. On solid electrodes •NO is oxidized to nitrosonium cation (NO^+^), which reacts with OH^−^ to produce NO_2_^−^ that is further oxidised to nitrate (NO_3_^−^). The instability of the compounds and the high overpotentials employed that are prone to interferences has lead to controversy on what exactly is measured by amperometry in brain tissue. On a carbon fiber microelectrode coated with a double layer of Nafion and o-phenylenediamine it was obtained a double wave oxidation of •NO (first at 0.9 V and the second at 1.2 V) and a single wave oxidation of NO_2_^−^ (at 1.2 V) and thus it was considered that at 0.9 V it can be detected only •NO [85]. Using a platinized carbon microfiber electrode at different values of potential (to +300, +450, +650, and respectively +850 mV) it was possible to detect the release of reactive oxygen and nitrogen species (•NO, NO_2_^-^, and ONOO^-^) from single cell (immunostimulated macrophages) [86].

In neurological research it is important to consider the effects of the anesthesia that may alter the concentration and evolution of the measured analytes. Due to practical limitations of the measurement devices, numerous experiments are performed on sedated animals with the cranium fixed in a stereotaxic frame. Comparable measurements in anesthetized and ambulatory animals demonstrated that it is not always possible to extrapolate the results from one state to the other. Different anesthetics have diverse effects on the same investigated analyte or physiological process. For example, the clearance of locally applied dopamine in anesthetized rats in comparison with awake animals is not significantly changed by urethane, but is inhibited by chloral hydrate that alters the dopamine transporter function [1]. The study of the drugs mechanism may be perturbed by anesthesia, e.g. urethane delays the effects of nomifensine (a psychostimultant that inhibits the dopamine uptake) and haloperidol (an antipsychotic that acts as dopamine receptor antagonist) on electrically evoked dopamine levels [87]. Not only the neurotransmitters are influenced by anesthetics, but also other physiological parameters like regional cerebral blood flow, oxygen and extracellular glucose levels. Oxygen is supplied by the blood and in consequence both parameters are directly correlated. For both blood flow and oxygen, it was observed that sodium pentobarbitone induced a decrease, ketamine produces a decrease followed by a rebound while chloral hydrate leads to an increase. All three anesthetics produced a decrease in extracellular glucose. The reduction of both glucose and blood flow produced by pentobarbitone suggests a reduction in glucose supply. The decrease in glucose coupled with an increase in blood flow induced by chloral hydrate hint to an increase in glucose utilization. The stress induced by anesthetics injection was assessed by the intraperitoneal injection of a saline solution and it was observed a brief increase in blood flow and oxygen accompanied by a decrease in extracellular glucose that does not interfere with the study of the long term effect of the anesthetics [88]. 

As a general observation, it is important to take into consideration that the response of enzymatic biosensors based on oxidases (like glucose oxidase or choline oxidase) depends on the oxygen concentration *in situ *and in consequence any oxygen variation induced for example by anesthetics or stress might affect the accuracy of the measured concentrations. The dependence of the micro-biosensor response on oxygen was demonstrated for the analysis of glutamate with microelectrodes based on glutamate oxidase. The post-mortem rise of glutamate levels was observed using microdialysis probes, but the micro-biosensors were not able to detect this variation exactly due to oxygen deprival [83].

## WIRELESS DEVICES

The alteration of “normal” brain chemistry induced by anesthesia and stress due to fixation constraints has lead to the development of wireless measuring devices. Despite the fact that wire connected microelectrodes allowed the real time monitoring of neurotransmitters even in the brain of living fish in water [89], the use of wireless measuring equipment has the advantage that the cable tethering is removed and thus the animal is completely free to interact with complex environment (going around obstacles) or have social contact with other animals (simultaneous investigation in more than one animal without the danger of cables getting tangled). This is possible by taking advantage of the advances in wireless devices in medicine (used to control the heart rate, blood pressure, ECG, etc.) and the general development in wireless communications (analog and digital broadcast, better Wi-Fi and Bluetooth standards, etc.). The first available equipment was analog, but subsequently were developed digital alternatives that have the advantage of lower noise, better reliability, higher transmission efficiency, security and timing. Nevertheless, the analog transmission has also its own advantages (cheaper, simpler, and smaller) and it is sufficient for many applications [90]. 

There are obvious equipment demands for a successful analytical implementation ranging from equipment miniaturization, low power demand or inductive charging, avoid of data cross-talk, speed of data transmission (higher for multielectrodes or high speed measurements), correct signal conversion, transmission and reception (modulation-demodulation process). An estimation of an usual data digital transmission rate for FSCV is: 1000 (measurement points/sweep)* 16 (bits for each point)/0.05 (seconds, the transmission time performed between cycles)= 320 kbits/s= 40 kBytes/s. The telemetry requirement for amperometry is much lower in comparison with FSCV: 2 (channels) * 1 (sample/second) * 16 (bits/sample) = 32 bits/s = 4 Bytes/s [91]. Even assuming a much higher measuring rate of 1000 measurements per second (seldom achievable due to the limitations imposed by the response time of the microelectrode/biosensor) the transmitted data remains much lower that FSCV: 32 kbits/s = 4 kBytes/s due to the longer transmission time. These calculations are related to the output signal, but two-way telemetry is necessary. The input data for FSCV (scan rate, scan type, upper and lower bias, repeat rate, gain, stimulus) is also much higher than for amperometry (only applied potential). These are theoretical calculations as the transmission implies data compression and packetization/balancing overhead [91]. The maximum data transfer rate is 1 Mbps for Bluetooth and 54 Mbps for Wi-Fi 802.11g, but the real rate depends on many particular parameters (distance, noise etc.). The reader should be aware that there are two different data units 8 bits = 1 Byte.

The microbiosensor devices can take advantage of the wireless devices developed for brain stimulation. An interesting example of wireless microelectrode used for brain stimulation is the steering of a rat in real time across a variety of terrains using stimulating electrodes implanted bilaterally in the somatosensory cortex coupled with another stimulating electrode implanted in the medial forebrain bundle for activating the brain reward system to train animals to turn in the appropriate direction depending upon whether the right or left somatosensory cortex was activated [92]. Until now there were developed several home-made proof-of principles of wireless equipment for FSCV measurements. One such demonstrative device based on Bluetooth transmission was used for dopamine analysis using a carbon fiber microelectrode and it was observed that the wireless device is less susceptible to noise in comparison with classical equipment that required a Faraday cage. The final optimized wireless device should weight ~17 g and could be attached on an adult rat using a backpack [45]. Dopamine and serotonin were measured by differential pulse voltammetry (DPV) or amperometry using a wireless device based on IR transmission that it is immune from electro-magnetic interferences that disturb radio-frequency trans-mission. The equipment weight is only few grams and uses only a single-way communication to avoiding cross-talking between two-way channels. It provided similar results for parallel *in vivo* experiments within anaesthetised rats with classical wire-connected equipment and was successfully applied to conscious freely moving rats [93]. 

## CONCLUSIONS

The vast information acquired in the last two decades using microbiosensors is invaluable for the understanding of numerous neurological processes. The continuous development of new and better measuring equipment and methods by researchers specialized in analytical techniques ready to be translated to neuroinvestigators provides the basis for future advancements in both theoretical and applicative brain exploration. Among future trends in microbiosensor development are the reduction of the probe size, the increase of the number of electrodes per probe leading to multianalyte detection or smaller overall structures, the simultaneous recording at different locations of one or multiple analytes (brain mapping), measurement without anesthetics in freely behaving animals using portable or wireless sensors. Microdialysis and microbiosensors must be considered as viable alternatives each one better suited for the investigation of a particular situation and not competing technologies in an evolutionary war destined to chose a single surviving technique. 

## Figures and Tables

**Fig. (1):**
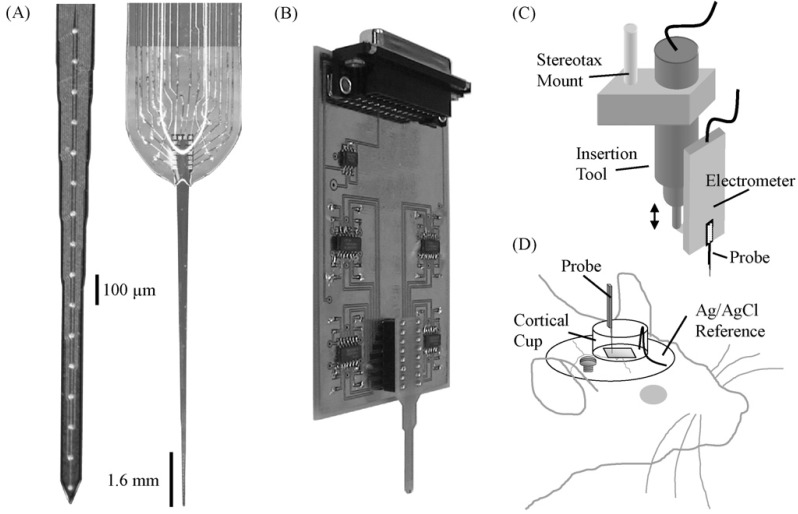
pH sensitive multielectrode array (**A**); custom 16-channel electrometer circuit with a 2×gain (**B**); insertion computer-controlled manipulator of a piezoelectric actuator used for the adjustment of the height of the electrometer and neural probe (**C**); cortical cup filled with phosphate buffer were the Ag/AgCl reference wire was placed (**D**). Reprinted from Journal of Neuroscience Methods 160, 276–287 (reference 18), Copyright (2007), with permission from Elsevier.

**Fig. (4):**
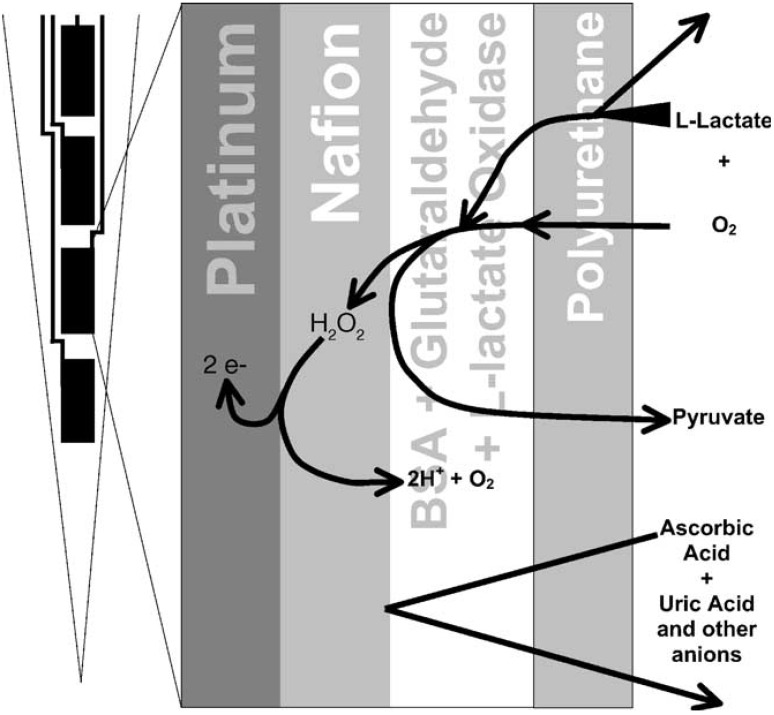
The working principle of L-lactate detection using microbiosensors based on L-lactate oxidase modified with two membranes: Nafion for interference removal and polyurethane for adjustment of linearity range. Please note that not all the hydrogen peroxide diffuses towards the electrode surface and a part of the anion interferents may reach the electrode surface. Reprinted from Biosensors and Bioelectronics 20(9), 1772–1779 (reference 67), Copyright (2004), with permission from Elsevier.
